# Fibrosis and Hepatocarcinogenesis: Role of Gene-Environment Interactions in Liver Disease Progression

**DOI:** 10.3390/ijms25168641

**Published:** 2024-08-08

**Authors:** Anindita Banerjee, Patrizia Farci

**Affiliations:** 1 Department of Transfusion Transmitted Diseases, ICMR-National Institute of Immunohaematology, Mumbai 400012, Maharashtra, India; anny.banerjee@gmail.com; 2 Hepatic Pathogenesis Section, Laboratory of Infectious Diseases, National Institute of Allergy and Infectious Diseases, National Institutes of Health, Bethesda, MD 20892, USA

**Keywords:** liver fibrosis, hepatocarcinogenesis, signaling pathways, fibrosis regression, genetic determinants, environmental factors

## Abstract

The liver is a complex organ that performs vital functions in the body. Despite its extraordinary regenerative capacity compared to other organs, exposure to chemical, infectious, metabolic and immunologic insults and toxins renders the liver vulnerable to inflammation, degeneration and fibrosis. Abnormal wound healing response mediated by aberrant signaling pathways causes chronic activation of hepatic stellate cells (HSCs) and excessive accumulation of extracellular matrix (ECM), leading to hepatic fibrosis and cirrhosis. Fibrosis plays a key role in liver carcinogenesis. Once thought to be irreversible, recent clinical studies show that hepatic fibrosis can be reversed, even in the advanced stage. Experimental evidence shows that removal of the insult or injury can inactivate HSCs and reduce the inflammatory response, eventually leading to activation of fibrolysis and degradation of ECM. Thus, it is critical to understand the role of gene-environment interactions in the context of liver fibrosis progression and regression in order to identify specific therapeutic targets for optimized treatment to induce fibrosis regression, prevent HCC development and, ultimately, improve the clinical outcome.

## 1. Introduction

The liver is one of the vital organs in the human body, with pleiotropic functions, including diverse metabolic roles and regulation of numerous physiological processes such as protein synthesis, energy metabolism, gluconeogenesis, glycogen storage, bile production, coagulation, detoxification, lipid regulation and others. As the liver microenvironment is constantly exposed to various endogenous or exogenous molecules and chemicals, it is vulnerable to injuries via several mechanisms, be it infectious, toxic, metabolic or immune-mediated. Importantly, the liver has an unlimited regenerative capacity compared to other human organs. However, persistent hepatic injury often gives rise to an aberrant and unbalanced wound healing response, causing chronic activation of hepatic stellate cells (HSCs) and unchecked extracellular matrix (ECM) accumulation, leading to hepatic fibrosis and cirrhosis. Normally, following activation, HSCs either undergo apoptosis or are inactivated by unknown regulatory mechanisms, and fibrolysis is activated. Whenever this balance is perturbed, uncontrolled fibrogenesis by persistently activated HSCs leads to progressive fibrosis, structural and functional alterations, and eventually cirrhosis and HCC. In advanced chronic liver disease and cirrhosis, liver transplantation is the only available treatment. Several lines of evidence indicate that fibrosis is the key driver of carcinogenesis in the liver. Although once thought to be irreversible, recent clinical studies show that hepatic fibrosis can be reversed, even in the advanced stage. Experimental evidence shows that removal of the insult or injury can drastically inactivate HSCs and reduce the inflammatory response, eventually leading to activation of fibrolysis and degradation of ECM [1,2,3,4]. Evidence has accumulated that antiviral therapy in chronic viral hepatitis patients results in substantial improvement of fibrosis, indicating that eliminating the underlying cause can halt and reverse, at least partially, liver fibrosis [1,3]. Nevertheless, despite effective antiviral therapy, in approximately 15–25% of viral hepatitis patients with compensated cirrhosis, liver fibrosis does not regress. A critical knowledge gap in the context of fibrosis regression is the characterization of the “point of no return”, i.e., the time point or stage whenceforth the liver cannot reverse the fibrosis. Moreover, not all patients show regression of fibrosis. Regression is probably not a linear process, and it may be influenced by the degree of fibrosis and its composition, as well as by genetic factors, other concomitant disease(s) and environmental factors.

From a clinical perspective, it is important to understand whether there are genetic determinants of fibrosis regression or progression and, if so, whether or not identification of these determinants can be utilized to optimize treatments that reduce liver fibrosis regression, prevent HCC development and, ultimately, improve the clinical outcome. A key question is which patients and at what stage they will show liver fibrosis regression. Can we predict who will regress and why? Is there a role of gene environment in controlling the fate of fibrosis? In light of recent technological advancements involving genomics and transcriptomic studies, certain pathways and genetic factors have been found to be associated with liver fibrosis dynamics. However, progress in the development of novel molecules/nanomedicine for anti-fibrotic therapy remains limited, with most studies restricted to early experimental trials to evaluate safety and efficacy. A deeper understanding of hepatic fibrosis is therefore needed to identify accurate markers and therapeutic targets for the development of effective anti-fibrotic drugs. This review aims to discuss the role of gene–environment interactions in the setting of liver fibrosis in the development of HCC and the crucial signaling pathways related to fibrosis and carcinogenesis.

## 2. Fibrosis Dynamics

The liver is a highly regenerative organ with a precise balance between ECM production and fibrolysis. Liver fibrosis results from dysregulated wound healing responses secondary to continuous and repeated liver injury induced by various viral and nonviral stimuli (metabolic or immunologic factors). Once considered as irreversible, accumulating experimental evidence demonstrates that fibrosis can regress and reverse to near normal [5].

The pathogenesis of liver fibrosis is complex, involving cross-talks among many signaling pathways, oxidative stress, metabolic regulation and immune responses. In the healthy liver, hepatocytes constitute 60–80% of the cellular population, and HSCs 5–15%. Normally, HSCs are in a quiescent state in the space of Disse and secrete hepatocyte growth factor (HGF), vascular endothelial growth factor (VEGF) and other soluble mediators [6]. HSCs also regulate the secretion of ECM proteins as well as the degradation of enzymes and the tissue inhibitors of these enzymes [7]. Cell death resulting from various infectious and non-infectious injuries, such as viruses, toxins, alcohol, and immunologic factors, is the initiating event that, in turn, leads to HSC activation and inflammation. HSC activation is the key event in fibrogenesis. Activated HSCs are transformed into myofibroblasts and continue to produce collagen and fibronectin [8]. This primary response, which forms a temporary fibronectin-rich ECM scar, acts as a scaffold for epithelial regeneration and protects against further hepatic injury. HSC activation also leads to the production of PDGF, which induces HSC proliferation in an autocrine manner [7]. HSCs also produce TGF-β, a potent inducer of collagen 1 production [9]. Meanwhile, invasion by inflammatory cells occurs to remove the debris while another inflammatory cascade induced by IL-6 and TNF-α leads to activation of Kupffer cells to replace hepatocytes [10]. In parallel, HGF and wingless-type MMTV integration site family member 2 (WNT2) signaling promote hepatocyte regeneration [10]. Macrophage recruitment following injury also promotes fibrogenesis. However, macrophages have contrasting functions, and they can also remove activated HSCs and increase matrix metalloproteinase (MMP) 12 and 13 expression to promote fibrosis resolution [11]. On the other hand, interaction between endothelial cells and HSCs in the space of Disse is also crucial as liver sinusoidal endothelial cells (LSECs) can actively repress the activation of HSCs [12]. Recent evidence suggests that LSECs can determine the fate of liver fibrosis. LSECs can promote hepatic regeneration through the expression of CXCR7 or can also result in persistent HSC activation and fibrosis via CXCR4 and FGFR-1 [13]. Thus, a highly concerted interaction and cross-talk among HSCs, macrophages, and LSECs through several inflammatory and signaling mediators ultimately restore the original hepatic architecture and balance. Upon repeated or persistent injury, however, transformed HSCs continue to produce the main components of the ECM and α-SMA, thereby promoting liver fibrosis. Invariably, HSC regulation is the essential factor driving scar tissue deposition and its lysis, while cellular cross-talk and immune landscape act in unison to define the phenotype [14,15]. As seen in chronically injured liver, lymphocytes and HSC localize in close proximity, suggesting functional interactions [16].

**Figure 1:**
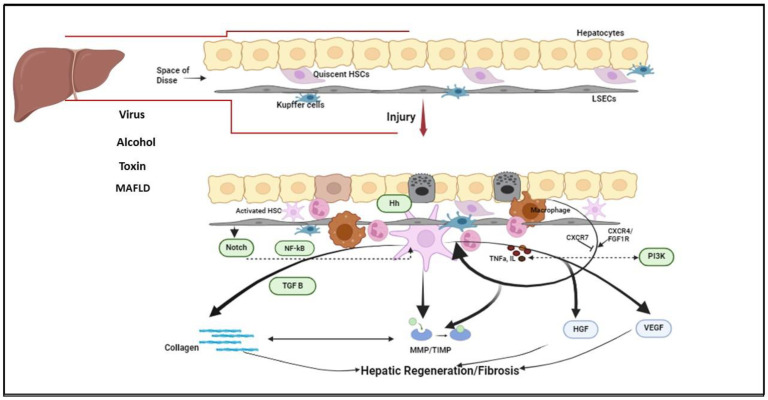
Pathogenesis and dynamics of liver fibrosis progression and regression. Hepatocyte injury leads to activation of inflammatory mediators and transformation of HSCs into myofibroblasts that, through an autocrine loop, aberrantly produce collagen alongside a disruption of the MMP/TIMP homeostasis. NF-kB, nuclear factor kappa B; TGF-β, transforming growth factor beta; Hh, Hedgehog pathway; IL, interleukin; TNFα, tumor necrosis factor alpha; PI3K, phosphoinositide 3-kinase; CXCR7, chemokine receptor 7; FGF1R, fibroblast growth factor 1 receptor; HGF, hepatocyte growth factor; VEGF, vascular endothelial growth factor; MMP, matrix metalloproteases; TIMP, tissue inhibitors of metalloproteinases. Image created in BioRender.com (accessed on 20 June 2024).

A central role in fibrolysis is played by MMPs, a zinc-containing, calcium-dependent group of proteases that can degrade the ECM tissue components [17]. Furthermore, MMPs have other regulatory roles in cellular proliferation, differentiation, migration, adhesion and apoptosis [18]. MMPS are in turn controlled by tissue inhibitors (TIMPS). MMP-1, 8 and 13 are the most potent proteases in the degradation of fibrillar collagen (Figure 1). MMP-1 plays an important role in inflammation and fibrosis pathogenesis. It can cleave both ECM constituent proteins such as collagen, laminin and gelatin, and non-ECM proteins such as complement C1q, IL-1β and TNF-α [19]. MMP-1 is constitutively expressed in human liver, mainly by the HSCs, and to different extents by Kupffer cells, monocytes and mast cells. MMP-1 can activate MMP-2 and MMP-9 [20,21]. Collagenases and gelatinases act in a sequential synergistic fashion to cleave collagen fibrils. MMP-1 and MMP-13 can cleave triple helical collagen fibrils into tropocollagen, which is again degraded by MMP-2 and MMP-9 [18,19].

Available reports indicate that MMP-1 overexpression alters the ECM network via the degradation of type-I collagen, leading to enhanced interaction between ECM and HSCs and hepatocyte proliferation, thereby promoting liver regeneration. It also aides in attenuating hepatic fibrosis by promoting HSC apoptosis [22]. Studies in fibrotic livers from patients with chronic viral hepatitis or alcoholic liver cirrhosis have found increased MMP-2 expression, which was associated with fibrosis severity [23,24]. MMP-9, known as gelatinase-B, can degrade type IV collagen, elastin, and fibronectin. MMP-9 is secreted by immune cells like neutrophils and macrophages, as well as by fibroblasts. Evidence shows that, during fibrosis and HCC development, ECM stiffness negatively modulates MMP-9 secretion and activity [25]. In contrast, a study by Prystupa et al. found high MMP-9 levels and activity in advanced alcoholic cirrhosis patients [26]. Thus, it appears that aberrant expression of different MMPs can promote fibrogenesis instead of scar degradation. However, while the majority of the studies are aimed at understanding the role of MMPs in liver disease progression, very few studies have focused on the role of MMPs during the resolution of liver diseases. Hepatic injury also affects the MMP–TIMP balance, leading to increased TIMP expression by activated HSCs, thereby inhibiting MMPs and scar degradation [19,27]. Upregulation of TIMP-1 and TIMP-2, along with diminished MMP1 expression, have been reported in patients with progressive liver fibrosis. Higher levels of liver and serum TIMP-1 have been found in patients as well as in animal models of liver fibrosis. Moreover, TIMP-1 expression was shown to be directly correlated with the stage of hepatic fibrosis [28]. In agreement with these findings, another study conducted among HCV and HIV/HCV-infected patients found higher TIMP-1 levels compared to those in healthy subjects, with the levels being positively correlated with liver stiffness [29]. An experimental animal study documented attenuation of liver fibrosis upon treatment with anti-TIMP-1 antibody [30]. In contrast, another study in TIMP-1-deficient mice could not confirm the role of TIMP-1 in liver fibrosis [28].

Two other critical mechanisms, namely, angiogenic response and HGF production by the HSCs, control the dynamics of hepatic regeneration. HSCs, through both direct and paracrine interactions with LSECs, synchronize vessel stabilization and sinusoidal remodeling via PDGF, TGF-β1, FGF and VEGF [31]. Deactivation and clearance of HSCs are essential regulatory mechanisms for re-establishment of the normal architecture and homeostasis of the liver. Activated HSCs can undergo apoptosis or become inactivated by unknown mechanisms. Recent evidence from studies in mice demonstrated downregulated expression of collagen type I alpha 1 chain (COL1A1), α-SMA and TIMP1 in inactive HSCs [32]. With regression, inflammatory infiltrates and oedema start disappearing from fibrous septa, making them thin and delicate, which helps in regenerating hepatocytes and leads to the eventual interruption of fibrogenesis. 

## 3. Major Signaling Pathways in Liver Fibrosis

Multiple signaling pathways play a major role in the pathogenesis of chronic liver diseases such as fibrosis, cirrhosis and HCC. Here, we will discuss the most important signaling pathways that have been reported to be involved in the pathogenesis of hepatic fibrosis and carcinogenesis, including NF-κB, transforming growth factor-β (TGF-β), PI3/Akt, Hedgehog, Notch and mediators of angiogenesis (Figure 1).

### 3.1. NF-κB

NF-κB signaling regulates the activation and apoptosis of HSCs through the regulation of mediators of inflammation such as TNF-α. Studies in rat models of liver fibrosis found upregulated expression of NF-κB signaling markers in the liver [33,34]. NF-κB signal transduction is mediated by both canonical and noncanonical signaling pathways. The canonical pathway involves IkB kinase (IKK), which leads to rapid but transient activation of target genes, while the noncanonical pathway is mediated by NF-κB-inducing kinase (NIK), leading to slow and persistent activation and inducible P100 processing [35]. Receptors for the noncanonical NF-κB signaling pathway belong to the tumor necrosis factor receptor (TNFR) superfamily. They include lymphotoxin beta receptor (LTBR), CD40 ligand, receptor activator for NF-κB ligand (RANK-L), B-cell-activating factor belonging to TNF family receptor (BAFFR) and OX40 [36,37,38]. The non-canonical NF-kB signaling pathway, under physiological conditions, is terminated by inactivation of NIK protein by ubiquitination and degradation mediated by TNF-receptor associated factor 2/3 (TRAF2/3) and cellular inhibitor of apoptosis 1/2 (cIAP1/2) [35]. Dysregulated noncanonical NF-κB signaling has been demonstrated in various liver diseases, including metabolic dysfunction-associated steatotic liver disease (MASLD), previously termed non-alcoholic fatty liver disease (NAFLD), alcoholic liver disease (ALD), autoimmune liver disease and viral hepatitis [35]. Transcription of TGF-β1 and other inflammatory cytokines is modulated by NF-κB [39]. The interaction of TGF-β1 and NF-κB signaling with lipopolysaccharide (LPS) was suggested to accelerate hepatic fibrogenesis [40]. One study by Wang et al. has shown that treatment with a barbiturate derivative in a CCL4-induced liver fibrosis model system blocked both TGF-β1 and LPS-induced NF-κB signaling pathways and inhibited HSC activation and macrophages recruitment and activation [41].

### 3.2. TGF-β/SMAD Pathway

The TGF-β/SMAD pathway plays a key role in hepatic fibrogenesis. It has been reported that activation of the TGF-β/SMAD pathway deposits excess ECM through enhanced TIMP1 expression, thus inhibiting MMP2 expression, and increases autophagy through the transcription of beclin [42,43,44]. Downregulation of NF-κB expression and nuclear translocation in Kupffer cells and HSCs lead to the inhibition of the TGF-β 1/p-SMAD3 pathway [39]. HSCs isolated from SMAD3 knock-out mice were shown to express less *COL1A1* mRNA, which is suggested to be mediated by p38 MAPK [45]. In addition, animal studies and a few studies from patients with liver fibrosis have shown that integrins and thrombospondin-1 (TSP-1) act as activators of TGF-β in advanced hepatic fibrosis, triggering a vicious cycle that eventually leads to cirrhosis [46,47].

Traditionally being considered as a profibrogenic cytokine owing to its role in HSC activation, TGF-β is also involved in hepatocyte proliferation, migration and regeneration through the modulation of other signaling pathways. Apart from SMAD, TGF-β receptors through alternative pathways (PI3K, MAPK, Ras and Rho-like small GTPases) can activate non-SMAD signaling responses in the liver [48]. Furthermore, studies after partial hepatectomy have shown that TGF-β is a critical regulator of hepatocyte regeneration [49,50]. An orchestrated interplay between TGF-β and tyrosine kinase receptor signaling triggered by the HGF or EGFR receptor determines hepatocyte proliferation and apoptosis during liver regeneration in a biphasic manner [48]. Several lines of evidence indicate that NADPH oxidases (NOX)-mediated ROS generation and downstream signaling play a significant role in hepatic fibrogenesis through the activation of HSCs mediated by TGF-β [51]. Evidence from both patients with chronic hepatitis C virus (HCV) infection and in vivo models of liver fibrosis showed upregulated levels of NOX4, increasing with the fibrosis degree [52,53]. Apart from being a master regulator of organ fibrosis, TGF-β is also implicated in epithelial to mesenchymal transition (EMT) [54]. Moreover, hypoxic factors, such as HIF-1α, can promote EMT in hepatocytes in a TGF-β dependent manner as a result of enzymatic activation of latent TGF-β by hypoxic hepatocytes [55]. TGF-β-induced EMT can be mediated both by SMAD and non-SMAD pathways, involving enhanced expression of E-cadherin transcriptional repressors and activation of cytoskeleton remodeling through ERK, respectively [56,57]. Interestingly, TGF-β has a dual role as a tumor suppressor in the early stage and a promoter in later stages, by mediating EMT and other mechanisms, as evidenced by several studies [58]. Activation of NOX1 by TGF-β promotes tumor cell proliferation in an autocrine manner through the activation of the EGFR pathway and NF-κB-mediated upregulation of EGFR ligand expression [59,60].

### 3.3. PI3K/Akt/mTOR Pathway

The PI3K/Akt pathway is an important signaling pathway regulating cell division and differentiation, autophagy and survival [61,62]. Platelet-derived growth factor (PDGF) is a strong chemoattractant of HSCs, and it stimulates the phosphoinositide 3-kinase (PI3K)/protein kinase B (Akt)/mammalian target of rapamycin (mTOR) signaling pathway [63]. Activation of the PI3K/Akt pathway induces anti-inflammatory cytokine expression and promotes an M2-like phenotype that facilitates tissue repair and resolution of inflammation [62]. Conversely, studies on animal models have shown that inhibition of the PI3K/Akt/mTOR pathway resulted in reduced hepatic inflammation and regulation of autophagy [64,65]. Recently, a study in a mouse model of liver fibrosis showed that tenofovir (TDF) treatment led to activated HSC apoptosis by downregulating the PI3K/Akt/mammalian target of the rapamycin (mTOR) signaling pathway, ultimately improving liver fibrosis [66]. This pathway is also actively involved in HCC initiation, metastasis and progression [67]. In normal cells, phosphatase and tensin homolog (PTEN) negatively regulates the PI3/Akt-mTOR pathway. However, PTEN is indirectly suppressed in HCC via activation of the PI3K pathway [68]. Therefore, modulation of the PI3K/Akt signaling pathway could be a potential effective therapeutic strategy against fibrosis and carcinogenesis.

### 3.4. Hedgehog Pathway

The hedgehog (Hh) signaling pathway has been demonstrated to play a critical role in HSC activation and liver fibrosis [69]. The hedgehog group of segment polarity proteins plays a critical role during embryogenesis by regulating cell proliferation, differentiation and tissue patterning [70]. Furthermore, it also has an important role in EMT as well as in the transformation of HSCs into myofibroblasts [71]. Experimental stimulation of sonic hedgehog (shh) in the liver in a transgenic mouse model was found to induce fibrosis and hepatocarcinogenesis [72]. While Hh ligands are minimally expressed in healthy liver, the ligands and Hh pathway activation are seen in any form of liver injury or insult [73]. Evidence shows that hedgehog signaling activation is proportionally linked to the severity of fibrosis and liver injury in NAFLD [74]. A recent study in CCL4-induced liver fibrosis in rats has found that the suppression of the Hh pathway by empaflifozin resulted in a reduction in the severity of fibrosis, possibly mediated through the inhibition of ER stress [75]. In addition, Hh signaling also promotes G2-to-M transition, resulting in tumor growth and proliferation [76].

### 3.5. Notch Pathway

The evolutionary conserved notch signaling pathway is involved in cellular communication and homeostasis, embryonic development and morphogenesis [77]. Furthermore, notch also acts as a tumor suppressor protein [77]. The notch pathway also acts in concert with other pathways such as TGF-β, FGF, Hippo, hedgehog and WNT signaling for cellular differentiation in the liver [78]. Although the role of notch is well understood in biliary regeneration, there is mixed evidence regarding its role in hepatocyte regeneration and fibrogenesis. Recent evidence in a rat model showed that the inhibition of notch led to impaired hepatocyte proliferation and cell cycle progression [79]. Studies in mice have shown that notch activation of LSECs can, in turn, activate HSCs, thus initiating fibrosis [80]. Another study in a mouse model of NASH has shown that hepatocyte-specific notch activation led to impaired HSCs activation and reduced fibrosis [81]. Moreover, experimental evidence indicates that notch mediates activation of the mTOR pathway, promoting lipogenesis [82]. Hepatotropic viral proteins also interact with notch signaling in different manners. Experiments in HBx stably transfected HepG2 cells (HepG2X) documented increased expression of cytoplasmic and nuclear notch proteins mediated by HBx protein through the p38 MAPK pathway [83]. Regarding HCV, the NS3 protein has been demonstrated to bind to Snf2-related CBP activator protein (SCARP) and p400, leading to activation of the notch transcriptional complex, which is involved in the modulation of immune responses in chronic HCV infection [84,85].

An abnormal notch signaling cascade plays a key role in the oncogenic transformation of hepatocytes, as well as in the proliferation and invasion of HCC [86]. Overexpression of HIF1α transcription factor is commonly found in HCC, and in vitro experiments found increased expression of several notch proteins like Notch 1, 2, 3 and 4 mediated by HIF1α [87]. Higher Notch1 and Jagged1 expression levels were found in HCC, where the metastasis grade was positively correlated with Notch1 mRNA levels [88].

### 3.6. Angiogenesis

Progression of liver fibrosis is closely associated with angiogenesis in fibrous septa. Primarily driven by hypoxia and inflammation, angiogenesis occurs in many organs after injury from various causes and is also a fundamental component of tumorigenesis and metastasis [89]. Therefore, it is interesting to evaluate it in the context of liver fibrosis and carcinoma development.

Persistent hepatic injury leads to activation of endothelial cells that, under the influence of VEGF, Ang-1, vb3 and vb5 integrins, accumulate in the form of tubular structures [90]. A fine orchestration of ECM deposition, MMPs, signaling pathways and neo-angiogenesis ultimately regulate the degree of vascularization on the background of fibrosis [91]. Continuous hepatic sinusoidal capillarization, together with progressive fibrosis, further diminishes the parenchymal oxygen supply, leading to upregulation of pro-angiogenic pathways through hypoxia in a vicious cycle [92]. HIF-1 signaling also activates the NF-κB pathway, thereby inducing inflammation [93]. Angiopoietin-1 (Ang-1) stabilizes newly formed vessels through Tie-2 receptor binding, while this effect is antagonized by angiopoietin-2 (Ang-2), which favors destabilization and vessel branching [94]. Aberrant Ang-2 expression has been observed in HCC. A study in tumorous and nontumorous hepatic tissues identified higher Ang-2 and lower Ang-1 expression in the tumorous tissue, suggesting that they play a role in angiogenesis associated with carcinogenesis [95]. Furthermore, the hepatitis B virus X protein (HBx) has been found to increase Ang-1 expression in the liver, which can be one of the mechanisms of HBV-induced carcinogenesis [96]. The drug sorafenib also inhibits angiogenesis through interaction with the ERK/MAP kinase pathway [97].

## 4. Fibrosis as the Chief Driver of Liver Carcinogenesis

Amidst several risk factors and mechanisms of liver carcinogenesis, a dominant role is played by hepatic inflammation, fibrosis severity and the liver microenvironment. More than 80% of liver cancers occur on the background of fibrosis [98]. Persistent liver injury, followed by hepatocyte death, activation of the inflammatory cascade and HSC activation, leads to increased oxidative stress and activation of various signaling pathways, all of which ultimately give rise to DNA damage, genomic alterations and oncogenic mutations [99]. Mutations of the genes involved in cell cycle, tumor suppression, mitogenic pathway and telomere regulation, e.g., *p53*, *TERT*, *RB*, *PTEN*, *CTNNB*, *CCNA2*, are observed in approximately 50% of HCC cases [100]. Upregulated angiogenesis also promotes tumorigenesis. PDGF, TGF-β, TNF-α, interferon and interleukins, particularly IL-1, IL-6 and IL-17 are major activators of HSCs and fibrosis [99]. Moreover, dysregulation of the immunologic environment, with reduced CD4+ T cells, increased regulatory T cells, immune exhaustion and impaired NK cell functions, further potentiate the development and evolution of liver tumors [101,102,103]. 

TGF-β-activated kinase 1 (TAK1) is a member of the MAPK kinase kinase (MAPKKK) family [104]. TAK1 plays an essential role in inflammation, cell proliferation, survival and metabolism [104,105]. TAK1 can rapidly be activated by TGF-β, which in turn promotes p38 and JNK activation [104]. The deletion of TAK1 in mouse hepatocytes was shown to cause fibrosis [106]. TAK1 also interacts with TGF-β in a positive feedback mechanism, where TGF-β in the ECM is complemented by the cellular loss of TAK1 [104]. 

Furthermore, alteration of the tissue microenvironment by fibrosis and cancer-associated fibroblasts (CAF) can influence liver cancer development [107]. α-SMA-positive myofibroblasts are activated HSCs that are found in both human and murine HCC. These myofibroblasts continue to deposit type-III collagen and laminin, while healthy liver mostly contains type-IV and type-VI collagen. This altered biomechanical environment facilitates aberrant cellular interactions, including integrins and DDR2 in hepatocytes and activation of related signaling pathways favoring tumorigenesis [108,109,110]. In contrast to several studies showing the integral role of HSCs and fibrosis in the development of HCC, an alternative hypothesis suggests that CAFs may limit cancer progression. Therefore, further studies are needed to investigate the possible tumor-suppressive functions of HSCs and CAFs in the context of liver cancer [111]. On the other hand, activation of MMPs in response to fibrosis also renders the liver microenvironment vulnerable to cancer development by releasing growth factors or generating cleavage fragments that can stimulate inflammatory and oncogenic signaling [16,112]. In addition, apart from driving carcinogenesis, the fibrotic environment also negatively influences the treatment outcome in HCC. Therefore, it is important to investigate whether the regression of liver fibrosis reduces the chance to develop HCC.

## 5. Can Genetic Markers Predict Fibrosis Outcome?

The advent of advanced sequencing technologies involving genomics and transcriptomics has been a true game changer in medical research. Such studies enabled us to identify the specific genetic variants or markers involved in either the development or severity of a wide range of diseases. However, given the wide variability of certain variants in different populations, those data need careful and detailed consideration before translation to clinical practice. Although there are several published GWASs findings from patients with different types of diseases, only a few specific variants have been identified to be universally affecting disease development. Likewise, for hepatitis C, researchers have delved deeper into identifying genetic markers and transcription factors implicated in the response to antiviral treatment, viral clearance and liver disease progression. Despite remarkable progress in decoding the important cells and pathways involved in hepatic regeneration, the impact of the host genetic factors in this process remains largely unknown. The notable genetic factors involved in liver fibrosis pathogenesis are discussed below.

### 5.1. IFN Lambda 4

The IL28B gene, located on the long arm of chromosome 19, encodes IFN-lambda3 (INF-λ3), which belongs to the type-III IFN family (IFN-λ). IFN λ and α proteins stimulate the ISG by JAK-STAT signaling pathways [113]. Growing interest in exploring the role of IFN λ in liver fibrosis has stemmed from the discovery of an association between IFN λ SNPs and major IFNL3/IFNL4 with HCV clearance [113]. Since then, several studies have looked at its role in liver fibrosis of other viral and nonviral etiologies and, interestingly, a similar association was found in chronic hepatitis B as well as in non-alcoholic fatty liver disease [114,115,116]. This association was further strengthened in two cohorts of 946 Italian patients with biopsy-proven NAFLD [117] and in an Anatolian cohort of 216 patients [118]. Another recent study conducted in chronic hepatitis C patients in Pakistan reported that *IFNL3-IFNL4* rs12979860 polymorphism could significantly predict hepatic fibrosis and cirrhosis [119]. These robust observations point out that fibrosis is the key determinant of long-term liver-related events [LRE], and thus the association of these outcomes with IFNL3/IFNL4 indicates a mechanistic link [120]. The HALT-C (Hepatitis C Antiviral Long-Term Treatment Against Cirrhosis) study conducted among 400 participants with approximately four years of follow-up for LRE found an increased number of adverse LREs, including hepatic decompensation and HCC, in participants with the rs12979860 major genotype compared to those with the minor genotype [121]. Despite these associations, the exact molecular mechanism of IFN-λ 3/4 contributing to inflammation and fibrosis regulation is yet to be defined. It is suggested to be mediated by alteration of the blood monocyte population and their migration, macrophage activation and increased chemotaxis of T cells [122,123].

Previous studies have shown that the chance of developing chronic HCV infection as well as a nonresponse to treatment are more likely in carriers of IFNL4-dG allele. Unexpectedly, the researchers found that the same alleles were associated with poor HCV clearance as well as with reduced risk of liver fibrosis [114,124]. A recent study has shown that IFN-λ4, by acting as a misfolded protein, causes persistent ER stress and apoptosis of hepatocytes and inhibition of HSCs proliferation [125].

### 5.2. Genetic Regulators of Apoptosis: RNF, MERTK, TULP

GWAS studies have found an association between hepatic fibrosis and markers of genetic regulation of apoptosis. One study in 2342 chronic hepatitis C patients found that SNPs in two functionally related gene, MERTK and TULP1(tub like protein 1), encoding factors involved in the macrophage mediated phagocytosis of apoptotic cells were associated with liver fibrosis progression [126]. Tub-like proteins (TULP 1 & 3) encode proteins involved in photoreceptor physiology and membrane trafficking [127]. TULP1 is also reported to be involved in phagocytosis pathways, DNA damage repair, and fibrosis [128]. Recent experimental findings demonstrated that TULP3 and SIRT1 can interact directly and regulate TGF-β mediated organ fibrosis [129].

On the other hand, MerTK, a member of the Tyro-Axl-MerTK (TAM) family of proteins, is highly expressed on macrophages. MerTK, via its multiple ligands, leads to engulfment of apoptotic cells, suppression of inflammation, synthesis of inflammatory resolution mediators and tissue repair [130,131]. In a study, the Rs6726639A variant, reported to be associated with lower hepatic expression of MERTK, was also associated with a reduced risk of liver fibrosis [132]. Thus, it indicates the possible role of MerTK in liver fibrosis [132]. Recently, a pathway of progression from steatosis to fibrosis in a NASH setting was suggested by the demonstration of a significant cross-talk between HSCs and liver macrophages through MerTK signaling leading to secretion of TGF-β1 [133].

GWAS studies also identified RNF7 (ring finger protein 7), which encodes a protein inhibiting reactive oxygen species (ROS) mediated apoptosis [126]. RNF7, also known as sensitive to apoptosis gene (SAG), belongs to SKP1-cullin/CDC53-F box protein ubiquitin ligases, as a subunit. RNF7 plays a role in the ubiquitin proteasome mediated protein degradation pathway, which is essential for cell turnover [134]. One study from an eastern European population found increased risk of cirrhosis in patients with RNF rs16851720 [135]. However, such associations with RNF7, MERTK and TULP1 SNPs were not found in a large Japanese GWAS of hepatic cirrhosis [136].

### 5.3. PNPLA 3

PNPLA3, a triglyceride lipase with weak transacylase activity, is predominantly expressed in hepatocytes and HSCs [137]. In hepatocytes, PNPLA3 is located on the surface of lipid droplets. PNPLA3 expression on hepatocytes is regulated by multiple metabolic and nutritional pathways, including the SREBP1c pathway [138]. Fasting decreases PNPLA3 expression, while refeeding or obesity is associated with high PNPLA3 expressions [139]. PNPLA3 plays a key role in HSC activation, and certain genetic variants in HSCs potentiate profibrogenic features [140]. Recently, Lindén et al. demonstrated the amelioration of NASH and hepatic fibrosis in human PNPLA3 I148M knock-in mice compared to wild-type mice, thus indicating an important role of hepatocyte PNPLA3 I148M as a potential target for intervention in liver fibrosis [141]. Furthermore, Pingitore et al. reported a protective effect of wild-type PNPLA3 in HSCs. They found that upregulated wild-type PNPLA3 in HSCs was associated with the reduced extracellular protein levels involved in fibrosis [142]. In contrast, a systematic review and meta-analysis determining the association between PNPLA3 rs738409 SNP and liver fibrosis severity, HCC risk and prognosis among patients with liver disease reported increased risk of advanced fibrosis. The study also found rs738409 to be an independent risk factor for HCC among patients with NASH or alcohol-related cirrhosis [143]. However, two recent studies in patients with chronic hepatitis C from Pakistan and Brazil did not find any significant role of PNPLA3 variants in modulating the development of hepatic fibrosis or cirrhosis in these patients [144,145]. Amidst these conflicting results, it is plausible that PNPLA3 induction occurs during HSC activation, but it does not necessarily lead to fibrogenesis, as shown by recent studies [146]. Consistent with this statement, recent data point to an inverse correlation between PNPLA3 expression and histological fibrosis scores [147].

### 5.4. TLL1

Tolloid-like 1(TLL1) encodes a metalloprotease of the peptidase M12A family. TLL1 is capable of cleaving procollagen C-propeptides, such as chordin, pro-biglycan and pro-lysyl oxidase [148]. The possible role of TLL1 has been studied in fibrosis and hepatocarcinogenesis. One GWAs in 456 Japanese patients with chronic hepatitis C identified TLL1 as a novel susceptibility locus for hepatocellular carcinoma (HCC) after HCV clearance following interferon- based treatment [149]. Consistent with this observation, clinical findings by John et al. showed that the TLL locus is also associated with fibrosis progression and is a risk factor for HCC development [150]. However, other cross-sectional studies in Latin American and European individuals found no evidence of the TLL1 rs17047200AT/TT genotype being a risk factor for HCC development [151]. Furthermore, studies conducted in European and Egyptian cohorts did not find any association between TLL1 polymorphisms and HCC occurrence in cirrhotic HCV patients after antiviral treatment [152,153]. Thus, the exact role of TLL1 in fibrosis progression and/or HCC development remains to be clarified.

### 5.5. TLR

Recently, increased knowledge on the role of pattern recognition receptors, including toll-like receptors (TLRs) in recognizing tissue injury through PAMPS (pathogen-associated molecular patterns in infectious condition) and DAMPS (damaged-associated molecular patterns in non-infectious condition) has influenced research into exploring their essential roles in tissue repair and organ fibrosis, like liver fibrosis [154]. Both hepatocytes and non-parenchymal cells express TLR4 in the liver. TLR4 signaling is involved in fibronectin production by HSCs and promotes fibrotic liver injury mediated by a variety of causative factors such as virus, alcohol, toxins, cholestasis, steatosis, autoimmune dysregulation and drugs. HSC-derived fibronectin also induces the migration of LSECs and angiogenesis [155]. Animal studies in liver fibrosis provide evidence for a role of TLR4 in the pathogenesis of liver fibrosis. A lower degree of liver fibrosis was documented in studies of carbon tetrachloride- or bile duct ligation-induced liver fibrosis in TLR4-mutant mice along with mutations in MyD88, CD14, TRIF and LBP [156,157,158]. One study found that TLR 4 enhances liver fibrosis by inducing TGF-β signaling [40]. A clinical study in chronic hepatitis C patients demonstrated that the TLR4 SNPs were associated with reduced risk of cirrhosis [159]. Subsequent studies found that TLR4 D299G and T399I SNPs were associated with diminished TLR4-mediated signaling and cell death in HSCs [160]. 

Apart from TLR4, there is some evidence about the involvement of other TLRs in liver fibrosis. Findings from a study by Miura et al. indicate that TLR9-mediated IL-1β signaling stimulates hepatocytes and HSCs to develop steatohepatitis and fibrosis in mice through MyD88, an adapter for TLR and IL1b [161]. A recent study by Zhou et al. showed that TLR5 activation induced by type-I IFN signaling protects against liver fibrosis by regulating the balanced production of IL-1β and IL1RN [162]. TLR5 agonist, CBLB502, has been shown to have protective effect against pulmonary fibrosis and pneumonitis in a radiation-induced mouse model [163]. Therefore, modulation of TLR5 signaling might be a promising target toward developing antifibrotic therapeutics for the improvement of liver fibrosis. In the context of HCV, experimental findings from human PBMCs indicate that HCV core and NS3 proteins activate TLR2/TLR1 and TLR2/TLR6 [164,165]. Similarly, HCV core protein activates TLR2 signaling to induce fibrogenic genes and MMPs in human HSCs, suggesting a direct role of HCV in liver fibrosis through TLRs [166]. Therefore, cross-talk between HCV viral proteins, TLR signaling and fibrogenesis pathways decides the final outcomes.

### 5.6. HLA DQ, HLA-E, HLA-C

Human leukocyte antigen (HLA) regions are important genetic markers for various diseases and their outcomes. Thus, several studies have explored the role of HLA loci variants in liver fibrosis in hepatology-related, genome-wide association studies. One study reported that Allele HLA-DQB1∗06 reduced fibrosis score in NAFLD patients [167]. Another study found that HLA-DQA1∗01 was independently associated with a reduced risk for NASH or NAFLD and pericellular and portal fibrosis, while HLA-DRB∗03 was observed to be associated with a higher risk for NASH NAFLD [168]. 

HLA-E, a nonclassical MHC class I molecule widely expressed in all human tissues, binds to natural killer cell receptors CD94/NKG2A, B and C [169,170]. HLA-E acts as a ligand for both innate and adaptive immune systems. Araujo et al. reported significantly higher HLA-E expression in the liver microenvironment in HCV-infected patients with severe fibrosis and necro-inflammation [171]. Thus, the nonclassical HLA-E molecule, through its interaction with immune cells and innate and adaptive immune system, could have a possible role in the severity of liver fibrosis, including its prognosis and outcomes. 

Another non-classical HLA molecule, HLA-G, known for its role in immune-tolerance modulation, could be involved in the differential outcome of liver disease. Studies in chronic hepatitis B and C patients showed that HLA-G is expressed in hepatocytes and biliary epithelial cells of the liver [172,173]. Moreover, 50.2% of the primary HCCs showed HLA-G expression with heterogenous staining, while it was not detectable in the adjacent non-tumor tissue [174]. These findings indicate the possible involvement of HLA-G in hepatic fibrogenesis and tumorigenesis. One study in HCV-infected liver samples found the presence of HLA-G in numerous cells in fibrosis septa, while it was not detected in hepatocyte nodules. Thus, HLA-G can serve as a putative marker of fibrosis as it is expressed by mast cells and may promote fibrosis by favoring a Th2 cytokine profile [175].

### 5.7. MICA

MHC class I polypeptide-related sequence A (MICA) is an interesting C-type lectin-like membrane glycoprotein, which is found to be overexpressed by infected, transformed, senescent and stressed cells in comparison to healthy normal cells, which barely express the protein [176,177]. MICA acts as a ligand for natural killer (NK) group 2D (NKG2D) [178]. MICA was identified from the first GWA study of HCV-related HCC in Japanese chronic hepatitis C patients as a SNP in the 5′ flanking region on chromosome 6, rs2596542, which was positively associated with the progression of chronic hepatitis C to HCC [179]. A study conducted in a large cohort of chronic hepatitis C patients with Caucasian ancestry demonstrated that *MICA* rs2596542 was associated with liver fibrosis progression. Another recent study found that *MICA* rs2596542 was associated with fibrosis progression, possibly mediated through TGF-β1-dependent mechanisms [180].

## 6. Role of Transcription Factors

Inactivation of HSCs, by either apoptosis or senescence, has been observed in experimental liver fibrosis models during the resolution of fibrosis. Several transcription factors (TFs) involved in maintaining the development, regulation or quiescence of HSCs, including GATA4/6, LHX2, TCF21 and ATF3, have been reported to be associated with HSC inactivation, while others, including PPARγ, GR, Elf-3, GLIS 2 and RARβ, have been involved in regulating the adipocyte-like state of HSCs and the genes targeting fibrosis-associated hepatokines [181]. Factors like ZNF469, TBX3 and RUNX1 have been reported to regulate fibrogenesis by HSCs [182]. It is becoming increasingly clear that TFs are often part of bigger interconnected networks governing gene expression in various cell types residing in the liver, leading to a vicious cycle of cytokine production and cross-talk among various cells, all of which act by promoting fibrogenesis. Thus, identifying suitable transcriptional regulators involved in fibrosis progression could reveal new approaches to treat liver fibrosis of various causes. 

Liver injury followed by unrestrained inflammatory response and progressive fibrogenesis is largely determined by the presence of other environmental factors influencing the liver microenvironment, leading to altered protein expression, dysregulated signaling cascade and histological changes. Globally, persistent alcohol abuse, smoking, metabolic dysfunction-associated fatty liver, hepatitis virus infections and exposure to certain drugs and toxins have been implicated in determining the final clinical outcome. Therefore, changes in the environment can either facilitate or prevent progressive liver fibrosis. Recent studies have identified key genetic and transcription factors implicated in liver fibrosis of different etiology. Table 1 lists some of the key factors associated with liver fibrosis.

### 6.1. TCF21

Transcription factor 21 (TCF21), also known as pod-1, capsulin or epicardin, encoded by the *TC21* gene on chromosome 6, is a ubiquitously expressed transcription factor involved in cell development, epithelial-mesenchymal transition and autophagy, and is implicated in carcinogenesis [197]. TCF21 has been identified as a deactivation factor for HSCs in murine models of hepatic fibrosis, thereby providing a potential therapeutic target for the otherwise intractable liver fibrosis [192]. The expression of TCF21 is decreased both in mice and human liver fibrotic tissue. One recent study has found that TCF21, through its downstream effector hnRNPA1, inactivates NF-kB signaling and promotes fibrosis resolution. The authors also reported that DNMT3a is responsible for the low expression of TCF21 in liver fibrosis [193].

### 6.2. ATF3

Activating transcription factor 3 (ATF3) is a transcription factor that plays pivotal roles in cellular response to inflammation, infection, ER stress, metabolic changes, and oncogenesis [185]. Studies in both humans and in rat models of MAFLD have shown that hepatic ATF3 protein induction promotes oxidative stress-mediated hepatic steatosis [185].

### 6.3. GATA 4/6 

GATA binding proteins belong to the GATA family of zinc finger transcription factors that bind to the ‘GATA’ motif, which is an important cis-element in the promoter region of numerous genes [198]. GATA4 is critical in embryonic and cardiac development and has also been implicated in hypertrophic cardiomyopathy [199]. This transcription factor has previously been shown to play an important role in embryonic HSC quiescence [200]. In a CCL4 mouse model of fibrosis, GATA overexpression in HSCs was shown to promote liver fibrosis regression [194]. Loss of GATA4 expression in hepatic sinusoidal cells induces liver fibrosis by increased sinusoidal capillarization and enhanced expression of profibrotic and angiogenic factors like PDGF, Sparcl1, Esm1 and Igfbp5 [195]. Transcriptional repression of HIF2α by GATA4 has uncovered the molecular mechanism of GATA4-mediated regression of fibrogenic HSCs [194]. While GATA4 is downregulated in hepatic fibrosis, GATA3 activation leads to HSC activation and fibrogenesis. Recent evidence has also pointed to a role of GATA3 in autophagy induction and HSC activation via regulation of the miR-370/HMGB1 pathway in a CCL4 mouse model of fibrosis [196].

### 6.4. ELF3 and GLIS2

A study by Loft et al. reported that knockdown of ELF3 and GLIS2 in a mouse model of liver fibrosis leads to altered expression of the hepatocyte biomarker Abcc4 and fibrosis-related signature genes, including Spp1 and Ctgf, contributing to HSC activation and liver fibrosis development [188].

### 6.5. RAR

HSCs also store retinoic acid, which can inhibit the fibrogenic process through activation of the retinoic acid receptor (RAR) [201]. PNPLA3 mutations tend to decrease the retinoic acid storage pools inside HSCs, leading to the reduction of RAR-mediated control on fibrogenesis [140].

### 6.6. PPARf

Peroxisome proliferator-activated receptors (PPARs) exert various biological and metabolic effects related to inflammation, atherosclerosis, adipocyte differentiation, glucose and lipid metabolism and carcinogenesis [202]. PPARs encompass three subtypes (PPAR-α, PPAR-β/δ and PPAR-γ) [201]. Available studies have reported a significant role of PPAR-γ in the improvement of hepatic steatosis, reduced inflammation and fibrosis and hepatitis virus clearance [203]. Over-expression of PPAR-γ coactivator-1α (PGC1α) in HCC cells led to inhibition of aerobic glycolysis by the inhibition of pyruvate dehydrogenase kinase isoenzyme 1 and prevention of subsequent cancer migration and invasion [204].

### 6.7. ZNF469

This transcription factor is a known regulator of ECM homeostasis and is implicated in rare disorders like Brittle Cornea Syndrome and Ehlers-Danlos syndrome [205]. Recent computational profiling of the transcription factors associated with liver fibrosis from 108 human liver biopsies matched with transcriptomes and epigenomes identified ZNF469 as a transcriptional regulator of collagen production in HSCs [182]. It can bind to and activate collagen genes type 1, 3 and 5. Experiments of targeted loss-of-function/inhibition of ZNF469 in HSCs will be important to confirm its role in liver fibrosis. 

## 7. Conclusions and Future Perspectives

Evidence from in vitro studies, animal models and epidemiological studies have revealed the key pathways involved in the molecular pathogenesis of HCC and its link with liver fibrosis. Specific genetic traits or transcription factors have been found to be associated with different clinical outcomes. However, there is a lacuna in identifying suitable prognostic or predictive markers that can identify patients that are most vulnerable to develop HCC. In addition, it is also critical to explore the role of gene-environment interaction in liver disease progression as well as regression. Several factors should be addressed for planning well-designed studies. Among the most important are the heterogeneity of fibrosis staging systems, the differences in cut-off values for liver stiffness measurement and the lack of adequate prospective studies looking at fibrosis dynamics. Despite current progress in experimental studies showing a number of promising candidates as antifibrotic drugs, only a few have progressed to early-phase clinical trials. Furthermore, there is an unmet need to assess the safety and efficacy of such potential candidates in liver fibrosis of different aetiologies. In addition, identifying molecules targeting multiple inflammatory or signaling pathways would be essential given the multifactorial pathogenesis of liver fibrosis. Considering innovative drug trials, either single or combination regimens might help to address the current loopholes and inaccuracies in terms of clinical effect. Therefore, it is urgent to identify predictive markers of liver fibrosis regression that in turn can be translated into clinical practice to develop potential antifibrotics to halt disease progression and restore liver histology. Furthermore, it will be critical to identify the proper timing at which these strategies can be implemented. 

## Figures and Tables

**Table 1:** Common environmental factors interacting with key genetic and transcription factors associated with liver fibrosis.

Environmental Factor	Genetic Factors Involved	Transcription Factors Activated/Suppressed
Alcohol	*PNPLA3*, *TM6SF2*, *MBOAT7*, *DRD2*, *CYP2E1* [183,184]	*ATF3* [185]
MAFLD	*PNPLA3*, *TM6SF2*, *MTTP*, *PPAR* [186] *COL1A1*, *IGFBP7*, *DCN*, *AEBP1*, *LGALS3BP*, *THBS2*, *FBLN2*, *FBLN5*, *FBN1*, *SERPINE1*, *ADAMTS2*, and *LUM* [182,187]	*LXR*, *FXR*, *SREBP*, *PPAR δ*, *ATF4*, *ELF3* and *GLIS2* *ZNF469*, *RUNX1* and *TBX3, ATF3* [182,185,188,189]
Hepatotrpic virus infection	*IL-28B*, *IFNL4*, *APOE*, *LDLr* *MMP-1*, *MMP-3* and *MMP9.* *TLR4*, *MTTP*, *MICA* [180]	*AP-1, PPARγ*, and *NF-κB* [190]
Animal models of liver fibrosis	*COL1A1*, *FBN1*, *BGN*, *COL6A3*, *MMP2*, *FBLN5*, *LUM*, *PDGFRB*, *LOXL1*, *SMAD2*, *SMAD4*, *YAP1*, *NOTCH1*, *EP300*, *p63*, *NCOR* [191]	*STAT3*, *NF-κB1*, *Sp1* *PPAR*, *HIF1*, *FOXO3*, *HDAC2*, *STAT5b* and *STAT6* [187] *TCF21* [190,192,193] *GATA 3*,*4* [194,195,196]

## Data Availability

The original contributions presented in the study are included in the article; further inquiries can be directed to the corresponding author.
